# Vision-related quality of life in patients with childhood-onset craniopharyngioma

**DOI:** 10.1038/s41598-023-46532-y

**Published:** 2023-11-10

**Authors:** Panjarat Sowithayasakul, Julia Beckhaus, Svenja Boekhoff, Carsten Friedrich, Gabriele Calaminus, Hermann L. Müller

**Affiliations:** 1 Department of Pediatrics and Pediatric Hematology/Oncology, University Children’s Hospital, Carl Von Ossietzky University, Klinikum Oldenburg AöR, Rahel-Straus-Strasse 10, 26133 Oldenburg, Germany; 2 Department of Pediatrics, Faculty of Medicine, Srinakharinwirot University, Bangkok, Thailand; 3 Division of Epidemiology and Biometry, Carl Von Ossietzky University of Oldenburg, Oldenburg, Germany; 4 Department of Pediatric Hematology/Oncology, University of Bonn Medical Center, Bonn, Germany

**Keywords:** Cancer, Paediatric cancer

## Abstract

Quality of life (QoL) is a critical component of aftercare in survivors of childhood-onset craniopharyngioma (CP). Visual impairment adversely affects QoL after CP. This study assessed the frequency of visual impairment in patients with CP and its association with QoL. This study analyzed vision-related QoL in patients recruited 2000–2019 in the prospective cohort studies KRANIOPHARYNGEOM 2000/2007. Ophthalmologic examinations were performed at diagnosis, three, 12, and 36 months, respectively after the diagnosis. The QoL (PEDQOL) scores, were also evaluated at three, 12, and 36 months, respectively after the CP diagnosis. Multivariable logistic regression was used to analyze factors associated with visual impairment during follow-up. One-hundred twenty patients were included in this study. On ophthalmological examination, visual impairment was observed in the majority of the patients (n = 84, 70%) at CP diagnosis. After surgery, vision was restored in 27 patients (32%) with visual impairment at diagnosis. In the first (*p* = 0.017) and third (*p* = 0.011) year after diagnosis, parents of patients with visual impairment reported lower social functioning (family). Reduced autonomy was found three years after diagnosis in self- (*p* = 0.029) and parental (*p* = 0.048) assessments. Next to visual impairment at diagnosis, no additional risk factors for visual impairment during follow-up could be identified. Visual impairment has a clinically relevant impact on QoL after CP. The visual status at CP diagnosis determines the visual outcome during follow-up. Early detection of visual impairment, regular QoL assessments, and risk-appropriate aftercare are recommended.

*Clinical Trial Registration* KRANIOPHARYNGEOM 2000 (Clinical trial registration number: NCT00258453) and KRANIOPHARYNGEOM 2007 (Clinical trial registration number: NCT01272622).

## Introduction

Childhood-onset craniopharyngiomas (CP) are malformational, embryonal tumors located in the sellar and parasellar area. The incidence of CP is 0.5–2 cases per million people per year^1,2^. Due to the benign histology of the tumor (WHO grade 1), a 10-year survival rate of 90% has been reported in children^3,4^. CP is one of the most challenging types of childhood central nervous system tumors due to the long-term sequelae caused by the proximity of CP to the optic nerves / optic chiasm and hypothalamic-pituitary axes^2,5–8^. Due to variability in tumor location, growth pattern, size, and suprasellar extension, CP patients present with different symptoms at the time of CP diagnosis^9^. Visual impairment is a primary manifestation in 62–84% of all patients at the time of CP diagnosis^10^. Defects in visual acuity (VA) and visual field (VF) are observed in CP patients due to the tumor itself or secondary to treatment. The postoperative visual outcomes following the surgical interventions for CP were influenced by a constellation of variables, such as tumor location and surgical techniques^11–14^. However, most of the existing studies had not focused on the visual outcomes; they were focused on the neurologic and endocrinologic prognosis^15^. Moreover, visual impairment in craniopharyngioma patients who underwent radiation therapy was a concern that required careful clinical attention. This was because radiation had the potential to harm the visual pathways, thus manifesting as radiation-induced optic neuropathy, retinopathy, visual pathway necrosis, and ocular toxicity^14,16,17^. For CP survivors, health-related quality of life (QoL) becomes a critical component of therapeutic outcome^18^. The hypothalamic syndrome results from disease- and/or treatment-related hypothalamic injury and leads to long-term severe sequelae compromising patient QoL^19,20^. Visual impairment adversely affects daily functioning and QoL in CP patients. Vision-related QoL encompasses an individual’s capacity to execute daily activities and engage in social interaction^17^. Moreover, the caregivers reported that blindness and visual impairment were the important health problems in over half of the CP survivors^21^. For this reason, our study analyzed the frequency of visual impairment at diagnosis and during follow-up and the vision-related QoL in patients with childhood-onset CP. Furthermore, we explored potential factors associated with the visual outcomes with the aim to establish valid benchmarks for future treatment approaches in CP patients.

## Materials and methods

### Patient characteristics

One hundred twenty patients (60 female / 60 male) diagnosed with childhood-onset, adamantinomatous CP were recruited in the multicenter, prospective observational cohort studies KRANIOPHARYNGEOM 2000/2007 between 2000 and 2019 to determine vision-related QoL. Eligibility criteria were age at diagnosis < 18 years, at least one ophthalmological examination during follow-up and complete information on treatment modalities (surgery and radiotherapy). Body mass index (BMI) was expressed as BMI SDS according to the reference values of Rolland-Cachera et al*.*^22^. The histological diagnosis of adamantinomatous CP was confirmed by neuropathological reference assessment in all cases.

### Neuroimaging

All preoperative and postoperative, axial, coronal, and sagittal magnetic resonance imaging (MRI) were reviewed by a reference neuroradiologist, blinded for clinical and surgical information, to assess tumor location, degree of surgical resection, preoperative hypothalamic involvement (HI), and surgical hypothalamic lesions (HL). As previously described^23,24^, HI of CP was categorized into defined degrees: grade 0 of HI: no detectable HI on preoperative MRIs; grade 1 HI: HI of the anterior hypothalamic area not involving mammillary bodies (MB) and hypothalamic structures dorsal of MB; and grade 2 HI: HI of both anterior and posterior hypothalamic structures, i.e. involving anterior hypothalamic area, MB and hypothalamic structures dorsal of MB^23,24^. Based on postoperative MRIs, postsurgical HLs were categorized according to the same criteria into three grades: grade 0 HL: no detectable HL on postoperative MRIs; grade 1 HL: HL of anterior hypothalamic structures not involving MB, and grade 2 HL: HL involving anterior hypothalamic areas, MB and hypothalamic structures dorsal of MB. The tumor size of CP was calculated using the formula “½ (A x B x C)” (aligned to the ellipsoid model: 4/3 π [A/2 × B/2 × C/2]), where A, B and C are the maximum dimensions in the standard planes: axial (transverse, A), coronal (cranio-caudal, B) and sagittal (antero-posterior, C) plane^24,25^.

### Ophthalmological examinations

Ophthalmological examinations performed at diagnosis, three, 12, and 36 months, respectively after the CP diagnosis were available for longitudinal analysis in 120 patients, who were included in our study on vision-related QoL after CP. All 120 patients underwent at least two ophthalmologic examinations, and 76 patients (63%) underwent four ophthalmologic examinations during the follow-up period. Visual acuity (VA) was assessed with a distance-projected Snellen Chart. Some children who could not be examined with the Snellen Chart were examined with the Tumbling E Chart. The tumbling E chart was performed in patients between 3–6 years of age^26^. All of the patients in our study were able to identify the orientation of the letter ‘E’. Fundoscopic examination was conducted and visual field (VF) examinations were performed by Goldmann Perimetry or computerized axial perimetry. Ocular motility was assessed by the Cover-Uncover Test. The Ishihara or Matsubara Color Vision Test was used for some patients. In our study, ‘visual impairment’ referred to any degree of vision loss that affected the participant’s normal daily life, which was defined by the inability to perform any activities that were generally considered to be routine or essential for independent functioning like eating and dressing^27^.

### Quality of life assessment

The multi-dimensional pediatric QoL (PEDQOL) questionnaire was used to evaluate the patients’ self-assessed and the patients’ parental-assessed QoL at 3, 12, and 36 months after CP diagnosis. The answers were grouped into seven domains: physical functioning, cognition, social functioning friends, social functioning family, and body image^28^. A high PEDQOL score is equivalent to more negative self- or parental-assessed QoL^28^. The parents’ rating QoL scores were not used as proxy indicators for CP patients’ QoL but rather as a measure of empathy. Beginning from an age of 4 years, patients were asked to self-evaluate their QoL using a pre-school version of the PEDQOL. Beginning with school-age (> 7 years), children received the school age questionnaire for self-assessment.

### Statistical analysis

Statistical analyses were performed using IBM SPSS statistic program version 24.0 and R version 4.2.1. Student’s *t*-tests were used for the comparison of normally distributed continuous variables between groups; Wilcoxon rank sum test for not-normally distributed continuous variables. Categorical variables were analyzed using Chi^2^ or Fisher’s Exact test (if at least one cell had an expected count of less than 5). A *p*-value ≤ 0.05 was considered statistically significant. A logistic regression analysis was conducted, both unadjusted and mutually adjusted, for the covariates to evaluate the association between radiation therapy, tumor location, tumor volume, degree of surgical resection, and visual impairment at diagnosis on the visual outcomes during the follow-up period. No adjustment for multiple testing was applied. Therefore, inferential statistics are intended to be exploratory (hypotheses generating), not confirmatory, and are interpreted accordingly. Missing values were handled using complete case analysis.

### Ethics approval

The study was conducted according to the guidelines of the Declaration of Helsinki and approved by the Ethics Committee of the Medizinische Fakultät, Julius-Maximilians-Universität Würzburg, Germany (140/99; 94/06).

### Consent to participate

Informed consent was obtained from all subjects (or their legal guardians) involved in this study.

## Results

A total of 120 patients (60 females / 60 males) with CP recruited in KRANIOPHARYNGEOM 2000/2007 between 2000 and 2019 were included in our study. This cohort of 120 patients was comparable with the total cohort of the KRANIOPHARYNGEOM 2000/2007 studies (Table 1).

**Table 1:** Characteristics of the study population of 120 patients diagnosed with childhood-onset, adamantinomatous craniopharyngioma (CP) and recruited in the studies KRANIOPHARYNGEOM 2000/2007 between 2000 and 2019. Categorical variables are presented as n (%).

Craniopharyngioma (CP) patient characteristics	Study cohort	KRANIOPHARYNGEOM 2000/2007
	n (%)	n (%)
Visual impairment at CP diagnosis	84 (70)	n.a
Sex, female/male	60 (50)/60 (50)	150 (50)/150 (50)
Median age at CP diagnosis, years (range)	10 (1.3–16.8)	8.8 (0.01–17.97)
First symptom in history of CP patients		
Headache	52 (42)	97 (32)
Visual impairment	24 (20)	58 (19)
Growth retardation	15 (12)	44 (15)
Nausea/vomiting	5 (4)	4 (1)
Polyuria/polydipsia	5 (4)	7 (2)
Neurological symptoms	5 (4)	19 (6)
Incidental finding	2 (2)	3 (1)
Precocious puberty	1 (1)	2 (1)
Weight gain	0 (0)	7 (2)
Other	0 (0)	9 (3)
n.a	11 (9)	50 (17)
Tumor volume cm^3^, median (range)	15.9 (0.9–202.5)	14.1 (0.002–637.1)
Tumor location		
Extrasellar	30 (25)	55 (18)
Intrasellar and extrasellar	89 (74)	202 (67)
Intrasellar	0 (0)	4 (1.3)
n.a	1 (1)	39 (13)

n.a. = data not available.

The median age at CP diagnosis was 10 years (range: 1.3–16.8 years). The most common presenting symptom at CP diagnosis was headache (n = 52, 42%), followed by visual impairment (n = 24, 20%) and growth retardation (n = 15, 12%). In 24 patients (20%), who were aware of visual impairment before the CP diagnosis, the history of the symptoms lasted for a median of 10 weeks (range: 1 week-36 months) before the CP diagnosis. On the ophthalmologic examination at the CP diagnosis, visual impairment was found in 84 patients (70%). In 24 patients who had visual impairment as the presenting symptom, 22 patients (92%) had visual impairment at diagnosis based on ophthalmologic examination. Visual acuity was less than 20/40 in one eye of 20 patients (17%) and in both eyes of 10 patients (8%). Visual field loss was identified in 39 patients (32%). Optic nerve atrophy was present at CP diagnosis in 30 patients (25%). Median initial tumor volume was 15.9 cm^3^ (range: 0.9–202.5 cm^3^). The tumor was located in both intrasellar and extrasellar regions in 89 CP patients (74%), and confined to the intrasellar region in 30 CP patients (25%) (Table 1). Median BMI SDS at the time of CP diagnosis was 0.94 SDS (range: − 3.09 to + 10.02). At CP diagnosis, 79 patients (66%) presented with HI^24,25^. Complete surgical resection was achieved in 25 CP patients (21%), whereas 95 CP patients (79%) underwent incomplete surgical resection. Seventy-one out of 95 patients (75%), who underwent incomplete resection, had visual impairment before surgery at the time of the CP diagnosis, whereas 19 patients (21%) who received incomplete resection, were presented without visual impairment at diagnosis (*p* = 0.02). The decision to perform a complete or incomplete resection of a CP was complex that involved multiple factors and often necessitated a multidisciplinary approach. Surgical HL^24,25^ were reference-confirmed by a neuroradiologist in 58 CP patients (48%). Sixty-one patients (51%) underwent postoperative irradiation (Table 2).

**Table 2:** Clinical characteristics of the study population of 120 patients diagnosed with childhood-onset, adamantinomatous craniopharyngioma (CP) and recruited in the studies KRANIOPHARYNGEOM 2000/2007 between 2000 and 2019 with or without visual impairment at diagnosis.

Characteristics	Overall	Visual impairment at dgx	No visual impairment at dgx	*p*
n, (%)	120 (100)	84 (70)	36 (30)	
Hypothalamic involvement (HI)^23^				0.27
Anterior HI	25 (21)	18 (82)	4 (5)	
Anterior and posterior HI	54 (45)	38 (72)	15 (13)	
No HI	7 (6)	7 (100)	0 (0)	
n.a	34 (28)	21 (18)	11 (10)	
Hypothalamic lesion (HL)^23^				
Anterior HL	32 (27)	21 (70)	9 (30)	0.24
Anterior and posterior HL	26 (22)	19 (73)	7 (27)	
No HL	33 (28)	27 (87)	4 (13)	
n.a	29 (24)	17 (59)	10 (3)	
Degree of surgical resection				
Complete resection	25 (21)	13 (52)	11 (44)	0.02
Incomplete resection	95 (79)	71 (75)	19 (20)	
Irradiation (XRT)	61 (51)	45 (78)	13 (22)	0.40
Photon irradiation	36 (59)	26 (76)	8 (23)	
Proton beam therapy	20 (33)	14 (74)	5 (26)	
Other XRT techniques	4 (7)	4 (100)	0 (0)	
BMI SDS^22^ at diagnosis				
Underweight (< − 2 BMI SDS)	5 (4)	1 (25)	3 (75)	0.15
Normal weight (− 2 to + 2 BMI SDS)	75 (64)	52 (74)	18 (26)	
Obesity (+ 2 to + 8 BMI SDS)	35 (30)	27 (77)	8 (23)	
Severe obesity (> + 8 BMI SDS)	3 (2)	2 (67)	1 (33)	
n. a	2 (2)	2 (100)	0 (0)	
Visual impairment three months after dgx	61 (51)	57 (93)	3(5)	< 0.001
n.a	20 (17)	13 (16)	4 (13)	
Visual impairment one year after dgx	65 (54)	57 (92)	5 (8)	< 0.001
n.a	14 (12)	10 (12)	3 (10)	
Visual impairment three years after dgx	54 (45)	49 (94)	3 (6)	< 0.001
n.a	20 (17)	15 (18)	4 (13)	

Categorical variables are presented with n (%). Dgx = diagnosis; n.a. = data not available; SDS = standard deviation score; IQR = interquartile range.

Longitudinal follow-up of the ophthalmological findings showed that three months after the CP diagnosis, visual impairment was still present in 57 out of 84 patients (68%). In 32% (n = 27) of the patients with visual impairment at diagnosis, no visual impairment was reported after surgery. Fifty-seven patients (68%) still had visual impairment after one year, and 49 patients (58%) after three years without aggravation (Table 2). Five patients without visual impairment at the CP diagnosis were presented with visual impairment one year after the CP diagnosis due to disease progression (Table 2). Three years after the CP diagnosis, information was missing in two of these patients and one patient had an improvement of the visual impairment (Table 2).

**Figure 1:**
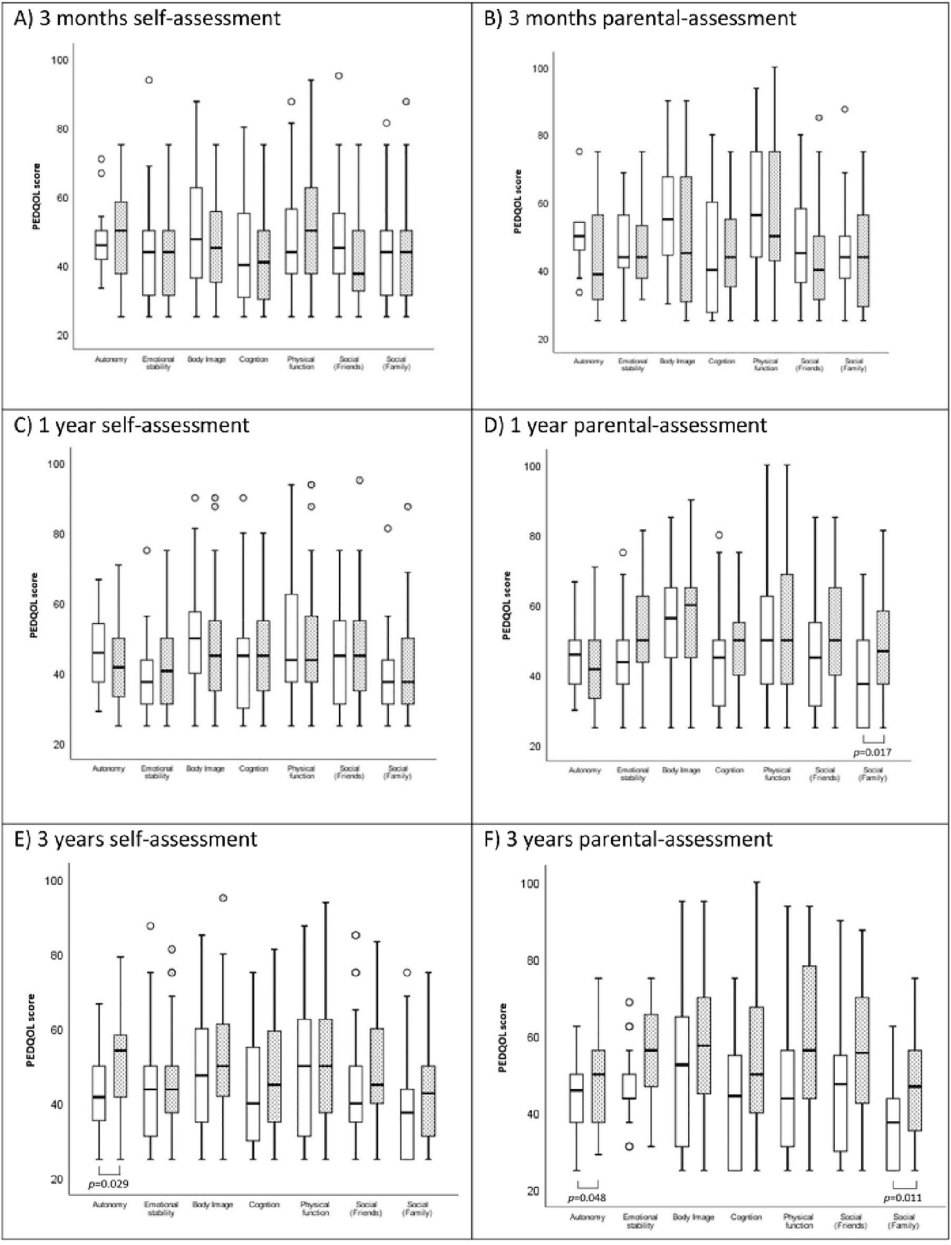
Self-assessment (**A**,**C**,**E**) and parental assessment (**B**,**D**,**F**) of quality of life by Pediatric Quality of Life Questionnaire (PEDQOL) in childhood-onset craniopharyngioma (CP) patients, recruited in KRANIOPHARYNGEOM 2000/2007, with regard to visual impairment (VI). White boxes: VI, and black boxes: no VI. PEDQOL scores are shown as negative rating at the time points three months (**A**,**B**), one year (**C**,**D**), and 3 years (**E**,**F**) after CP diagnosis. The horizontal line in the middle of the box depicts the median. The top and bottom edges of the box respectively mark the 25th and 75th percentiles. Whiskers indicate the range of values that fall within 1.5 box-lengths. Outliers (values beyond the 75th or below the 25th percentile) are marked as bullets.

The multi-dimensional PEDQOL questionnaires of patients’ self- and parental-assessed QoL were analyzed at 3, 12, and 36 months after CP diagnosis (Fig. 1). At the third month after CP diagnosis, no differences in the PEDQOL domains between CP patients with and without visual impairment were observed. At 12 months after CP diagnosis, a difference was found in parental assessment of the domain of social functioning in familial context between patients with and without visual impairment (*p* = 0.017). At 36 months after CP diagnosis, differences of the self- and parental-assessed autonomy score were identified comparing the CP patients with and without visual impairment (*p* = 0.029 and *p* = 0.048, respectively). Moreover, a difference of the parental-assessed social familial functioning score was observed at the 36th month after CP diagnosis (*p* = 0.011).

Logistic regression analyses were performed to identify potential risk factors of visual impairment after one and three years post-diagnosis in CP patients including the following parameters: radiation therapy, tumor location, tumor volume, grade of surgical tumor resection, and visual impairment at CP diagnosis. Incomplete surgical resection was not statistically significant associated with visual impairment (OR = 2.58, 95% CI: 0.98–7.00) one year after CP diagnosis. But after adjusting for visual impairment at diagnosis, tumor location and tumor volume, the OR decreased (OR = 1.39, 95% CI: 0.39–5.03). None of the other investigated parameters were statistically significant associated with visual impairment one year after CP diagnosis (Table 3). After three years post-diagnosis, no statistically significant associations were found but the effect of incomplete surgical resection (OR = 1.39 95% CI 0.51–3.85) was reversed after adjusting for visual impairment at CP diagnosis, tumor location and volume (OR = 0.54, 95% CI 0.11–2.15)(Table 4). Nonetheless, the visual outcome after one (OR = 16.52 95% CI 5.25–65.11) and three (OR = 24.25 95% CI 6.49–133.76) years is mainly dependent on the initial visual status at diagnosis (Tables 3, 4).

**Table 3:** Results of univariable and multivariable logistic regression on visual impairment (yes/no) one year after diagnosis of childhood-onset, adamantinomatous craniopharyngioma (CP) in 120 patients recruited in the studies KRANIOPHARYNGEOM 2000/2007 between 2000 and 2019.

Characteristics	Unadjusted OR	95% C.I		*p* value	Adjusted OR	95% C.I		*p* value
		Lower	Upper			Lower	Upper	
Radiation therapy (in the first year after CP dgn)	1.35	0.35	4.87	0.65	–	–	–	–
Intra- and extrasellar tumor location	1.96	0.81	4.77	0.13	1.01	0.30	3.13	0.99
Tumor volume	1.01	1.00	1.04	0.20	1.01	0.99	1.04	0.64
Incomplete surgical resection	2.58	0.98	7.00	0.06	1.39	0.39	5.03	0.60
Visual impairment at diagnosis	15.96	5.65	53.52	< 0.001	16.52	5.25	65.11	< 0.001

CP = craniopharyngioma; C.I. = confidence interval; OR = odds ratio.

**Table 4:** Results of univariable and multivariable logistic regression on visual impairment (yes / no) 3 years after diagnosis of childhood-onset, adamantinomatous craniopharyngioma (CP) in 120 patients recruited in the studies KRANIOPHARYNGEOM 2000/2007 between 2000 and 2019.

Characteristics	Unadjusted OR	95% C.I		*p* value	Adjusted OR	95% C.I		*p* value
		Lower	Upper			Lower	Upper	
Radiation therapy (in the first 3 years after CP dgn)	1.32	0.24	6.7	0.73	–	–	–	–
Intra- and extrasellar tumor location	1.42	0.6	3.49	0.43	0.97	0.28	3.09	0.96
Tumor volume	1.02	1.00	1.05	0.11	1.01	0.99	1.05	0.34
Incomplete surgical resection	1.39	0.51	3.85	0.52	0.54	0.11	2.15	0.41
Visual impairment at diagnosis	19.17	5.87	87.40	< 0.001	24.25	6.49	133.76	< 0.001

CP = craniopharyngioma; C.I. = confidence interval; OR = odds ratio.

Since only a minority of the included patients received radiation therapy during the first year (n = 42) or during three years (n = 54) after CP diagnosis, irradiation as a parameter was not assessed in the final model (Tables 3, 4).

## Discussion

Our study investigated the association between visual function and health-related QoL and the possible risk factors of visual impairment in CP patients. In children with CP, the main initial symptoms result from increased intracranial pressure causing headache and compression of the visual pathway with consecutive visual impairment. In our study, the two major presenting symptoms at the time of CP diagnosis were headache and visual impairment, which were observed in 70% of patients at the time of CP diagnosis. The most common fundoscopic findings at the time of CP diagnosis included decreased VA, VF loss, and optic nerve atrophy at 25%, 32%, and 25%, respectively. Our findings support previous reports on initial ophthalmological findings in CP patients^1,5,11,29^. Interestingly, the prevalence of self-reported visual impairment at diagnosis was lower than the prevalence found with the ophthalmologic examination which supported the previous study about the lack of the ability to articulate visual loss effectively and describe abnormal visual symptoms clearly in children^30^. The visual improvement post-surgical resection of a CP could be attributed to multiple factors that contributed to the alleviation of the pressure or damage to the visual pathway, particularly the optic chiasm, optic nerves, and optic tracts^12^.

We found a lower parental assessment of the patient’s QoL in the social-family domain in the first and third year after CP diagnosis in patients with visual impairment. Furthermore, in the third year after CP diagnosis, QoL in the autonomy domain assessed by patients and parents was lower in patients with visual impairment. We suggest these might be due to the focus on the overall health overshadowing the impact of the visual impairment on QoL leading to no observed differences in the third month. Over time, the cumulative effects of visual impairment would become more pronounced, thus affecting social functioning and autonomy, as evident at 12 and 36 months, respectively. Additionally, both the patients and parents may take time to fully recognize the impact of the visual deficits leading to the observed QoL differences at later stages. In terms of potential risk factors for visual impairment at the follow up, no statistically significant associations were found based on our data.

Tumor- and treatment-related sequelae affect QoL in survivors of CP^31^. In previous studies, younger age at CP diagnosis and preoperative functional impairments, large tumor volume, and hypothalamic / third ventricle involvement at initial presentation were reported as important factors that negatively affected patients’ QoL^24,32–35^. Due to the crucial importance of hypothalamus-sparing surgical strategies^36^, most discussions on QoL after CP are dealing with neurologic and endocrinologic sequelae and outcome, rather neglecting visual impairments that affect health-related QoL of pediatric brain tumor survivors’ and their families^37^. At least in part due to age-dependent difficulties of conducting an eye examination in pediatric patients, visual dysfunction and decreased vision-related QoL are often not recognized in these patients^17^.

With regard to longitudinal follow-up, pediatric brain tumor survivors with visual impairment have been reported to develop significant behavioral, internalizing and social problems^38^. Social difficulties were also noted in pediatric survivors of CP^29,39^. In our longitudinal study, the social and autonomy domain of QoL were affected in patients with visual impairment. During follow-up, the difference in the PEDQOL social life domain was observed by parents after one and three years, not by patients themselves, indicating the different perspective on the own social and family life. A reduced autonomy of CP patients with visual impairment was reported three years after diagnosis in self and parental assessment, showing the longitudinal influence of visual impairment on the personal development of the children. Accordingly, we suggest to evaluate and analyze vision-related QoL not only at the time of CP diagnosis but also longitudinally during follow-up.

Visual impairment can be caused by CP therapy, especially surgical resection, via direct surgical trauma of the optic pathway or perioperative visual loss^40^. Hence, in patients with a large tumor size or tumor location close to vital structures such as the optic chiasm / nerves and the hypothalamus, partial resection sparing critical structures is currently recommended as the treatment of first choice^41^. However, the advantages of limited tumor resection versus gross-total resection are still discussed controversially in the literature^42–46^. Wan et al. identified next to younger age at diagnosis (< 10 years), tumor recurrence and optic nerve edema as risk factors for visual decline; surgical resection was not statistically significant associated with visual decline^13^. Sughrue et al*.* reported a worse visual outcome in CP patients receiving irradiation after subtotal tumor removal compared to total tumor removal or subtotal tumor removal alone^47^. Fisher et al*.* also reported that the degree of surgical resection was not associated with the postoperative visual outcome^4^. Nonetheless, these observations were strongly contested by Hetelekidis et al*.* who showed that visual loss was associated with aggressive surgical strategies^3^. When patients presented in our study with visual impairment at the time of CP diagnosis, clinically significant improvement in their vision after surgery was observed in 32% of patients with visual impairment at diagnosis. Based on our study, the risk of visual decline after surgery for patients with normal visual function at the time of CP diagnosis is small. The visual status is mostly stable after surgical intervention.

In our analyses, incomplete surgical resection demonstrated unadjusted a tendency for visual impairment after three years (OR = 1.39, 95% CI 0.51–3.85), although this tendency was not statistically significant. Based on our multivariable analyses, we propose that incomplete surgical resection might have a rather protective effect (OR = 0.54, 95% CI 0.11–2.15) on the risk of developing long-term visual impairment after CP compared to total surgical resection, when potential covariates as tumor volume and location are considered. Especially in patients presenting with visual impairment, a hypothalamus-sparing surgery is recommended. Moreover, we would speculate that large tumor volume and consecutive pressure on neighboring structures, such as the optic nerves and optic chiasm, are risk factors for initial visual impairment. These pre-diagnostic risk factors are not modifiable. However, if these factors are initially diagnosed, a surgical tumor volume reduction and relief of pressure on the optic tract are the treatments of choice and are mostly performed as emergency interventions in order to salvage visual function. Further prospective observational studies with larger sample size and longer follow-up are needed to draw definite conclusions on the risk of surgical strategies on visual impairment in CP patients.

With regard to irradiation, a previous study suggested that subtotal tumor removal followed by adjuvant radiotherapy could provide tumor control rates in CP similar to those for total tumor removal^48^. However, the risk of visual impairment after irradiation remained as a major argument against adjuvant radiation in this region. Likewise, previous studies reported that patients who received radiotherapy experienced an increased rate of visual compromise compared to patients who underwent surgery only^47,49^. Although management of CP remains controversial, our study revealed a tendency towards a benefit of limited surgery under protection of critical structures.

Our study has certain strengths and limitations. The results of our study were limited due to missing data in the clinical information and the low proportion of patients who received radiotherapy during the first three years after the CP diagnosis. Accordingly, no conclusion on the influence of radiotherapy on the risk of visual impairment could be made. Furthermore, the assessment period of three years post-diagnosis was too short to evaluate the long-term visual consequences of radiotherapy. An assessment after several years post radiotherapy might be useful to evaluate the long-term consequences of the treatment. Moreover, the small sample size of our study added another layer of complexity for interpreting the results. Small sample sizes are prone to various statistical issues, including multiple testing errors and broad confidence intervals in multivariable analyses. This would further challenge the reliability and generalizability of the findings, particularly when attempting to discern associations between the incomplete resection and visual outcomes. Regarding the ophthalmological screening at diagnosis, the discrepancy between "self-reported visual impairment" and the objective findings from the ophthalmological examinations are noteworthy. Given these disparities, we recommend that comprehensive ophthalmological evaluations be integrated as a standard component at the time of diagnosis. This would not only provide a more accurate baseline for the visual function, but also aid in the longitudinal monitoring of the potential changes in visual acuity or fields, thereby informing tailored treatment strategies. Although our study focused on the subject of the impact of visual impairment on QoL, it would be essential to recognize that CP survivors often face a multitude of other challenges, such as endocrine dysfunction, neurocognitive effects, and psychosocial issues, that could independently or synergistically influence QoL.

Anyhow, our study had several strengths. Our study was a prospective longitudinal study performed at standardized follow-up intervals. Although pediatric CP is a rare condition, we were able to include 120 patients in this analysis. We also examined both, self- and parental-reported QoL, in CP patients, which is important to evaluate both perspectives on the social life of the families.

In conclusion, visual impairment is one of the most frequent symptoms of CP in children. Patients with normal visual function at the time of CP diagnosis can expect good visual outcome after hypothalamus-sparing surgical interventions. Restoring visual function after surgery was achieved in 32% of patients with visual impairment at diagnosis. Survivors of childhood-onset CP with visual impairment developed poor behavioral outcomes for social functionality in the family and with regard to autonomy. Thus, regular assessment of patients’ QoL and appropriate psychosocial support should constitute an integral element of follow-up care in CP tumor survivors. Further research in prospective observational cohort studies is needed to identify potential risk factors for long-term visual impairment.

## Data Availability

The datasets used and/or analysed during the current study available from the corresponding author on reasonable request.

## Abbreviations

BMI: Body mass index
CP: Craniopharyngioma
HI: Initial hypothalamic involvement
HL: Surgical hypothalamic lesion
MB: Mammillary body
MRI: Magnetic resonance imaging
OR: Odds ratio
PEDQOL: Multi-dimensional pediatric quality of life questionnaire
QoL: Quality of life
VA: Visual acuity
VF: Visual field
VI: Visual impairment
WHO: World Health Organization
